# CAZyme prediction in ascomycetous yeast genomes guides discovery of novel xylanolytic species with diverse capacities for hemicellulose hydrolysis

**DOI:** 10.1186/s13068-021-01995-x

**Published:** 2021-07-02

**Authors:** Jonas L. Ravn, Martin K. M. Engqvist, Johan Larsbrink, Cecilia Geijer

**Affiliations:** 1 Department of Biology and Biological Engineering, Chalmers University of Technology, 412 96 Gothenburg, Sweden; 2 Wallenberg Wood Science Center, Chalmers University of Technology, 412 96 Gothenburg, Sweden

**Keywords:** Ascomycota, Non-conventional yeasts, CAZymes, Xylanase, Xylan, Xylanolytic yeasts

## Abstract

**Background:**

Ascomycetous yeasts from the kingdom fungi inhabit every biome in nature. While filamentous fungi have been studied extensively regarding their enzymatic degradation of the complex polymers comprising lignocellulose, yeasts have been largely overlooked. As yeasts are key organisms used in industry, understanding their enzymatic strategies for biomass conversion is an important factor in developing new and more efficient cell factories. The aim of this study was to identify polysaccharide-degrading yeasts by mining CAZymes in 332 yeast genomes from the phylum Ascomycota. Selected CAZyme-rich yeasts were then characterized in more detail through growth and enzymatic activity assays.

**Results:**

The CAZyme analysis revealed a large spread in the number of CAZyme-encoding genes in the ascomycetous yeast genomes. We identified a total of 217 predicted CAZyme families, including several CAZymes likely involved in degradation of plant polysaccharides. Growth characterization of 40 CAZyme-rich yeasts revealed no cellulolytic yeasts, but several species from the Trichomonascaceae and CUG-Ser1 clades were able to grow on xylan, mixed-linkage β-glucan and xyloglucan. *Blastobotrys mokoenaii, Sugiyamaella lignohabitans*, *Spencermartinsiella europaea* and several *Scheffersomyces* species displayed superior growth on xylan and well as high enzymatic activities. These species possess genes for several putative xylanolytic enzymes, including ones from the well-studied xylanase-containing glycoside hydrolase families GH10 and GH30, which appear to be attached to the cell surface. *B. mokoenaii* was the only species containing a GH11 xylanase, which was shown to be secreted. Surprisingly, no known xylanases were predicted in the xylanolytic species *Wickerhamomyces canadensis*, suggesting that this yeast possesses novel xylanases. In addition, by examining non-sequenced yeasts closely related to the xylanolytic yeasts, we were able to identify novel species with high xylanolytic capacities.

**Conclusions:**

Our approach of combining high-throughput bioinformatic CAZyme-prediction with growth and enzyme characterization proved to be a powerful pipeline for discovery of novel xylan-degrading yeasts and enzymes. The identified yeasts display diverse profiles in terms of growth, enzymatic activities and xylan substrate preferences, pointing towards different strategies for degradation and utilization of xylan. Together, the results provide novel insights into how yeast degrade xylan, which can be used to improve cell factory design and industrial bioconversion processes.

**Supplementary Information:**

The online version contains supplementary material available at 10.1186/s13068-021-01995-x.

## Background

Revolutionizing the use of biomass is one of the most promising pathways to a more sustainable production of liquid fuels, chemicals and materials and a reduced fossil fuel dependence. The global benefits of a ‘green shift’ towards a circular, biobased economy are numerous and include lower CO_2_ emissions, resilient product and food chains and creation of stimulating high-skilled jobs [1]. However, for it to be realized, many technological hurdles and biochemical challenges in waste minimization and resource conversion efficiency must be overcome [1, 2].

Lignocellulosic biomass is mainly composed of the homopolysaccharide cellulose (40–60% of dry weight), various hemicellulosic heteropolysaccharides (20–35% of dry weight), and the aromatic polymer lignin (15–40% of dry weight) [3]. Cellulose is a linear polysaccharide consisting of β-1,4-linked d-glucose units that form crystalline and insoluble microfibrils [4]. Hemicelluloses coat the cellulose fibrils and their proportions and abundances differ between plant species. In industrially important grasses and hardwoods, xylans are the most abundant hemicellulose type, while in other species galacto-glucomannans, xyloglucans and mixed-linkage β-glucans are more abundant [5–7]. Xylans comprise a backbone of β-1,4-linked d-xylose residues which are commonly *O-*acylated and further substituted by α-1,2- or α-1,3-linked arabinosyl units and α-1,2-linked (methyl)-glucuronic acid moieties, and these carbohydrate decorations can in turn be further substituted in various patterns. The xylans are typically grouped into arabinoxylan (AX), glucuronoxylan (GX) and glucuronoarabinoxylan (GAX) [8]. The arabinosyl substitutions found on xylans can be esterified with ferulic acid that can in turn form phenolic crosslinks to other feruloylated xylans or the hydrophobic lignin polymers in the plant cell wall [9], thereby exerting biomechanical contributions to cellulose fibrillar networks [10]. For biorefining purposes, the complex and heterogenous carbohydrate matrix in the plant cell wall represents one of the main challenges in efficient and rapid conversion of biomass and biowastes to value-added chemicals and fuels [11, 12].

Microbes and their carbohydrate-active enzymes (CAZymes) are central for depolymerization of the complex lignocellulosic polysaccharides in the global carbon cycle as well as in industrial bioconversion processes [13]–[15]. Complete or semi-complete enzymatic breakdown of biomass requires multiple exo-, endo- and auxiliary CAZymes to hydrolyze the diversity of polysaccharide backbones and side chains [16, 17]. CAZymes are divided into classes and families in the carbohydrate-active enzymes database (CAZy, www.cazy.org; [18]) based on their sequence similarities, which in turn determine their structures and functions, e.g., catalysis reactions [19]. The enzyme classes in CAZy comprise glycoside hydrolases (GHs), glycosyl transferases (GTs), polysaccharide lyases (PLs), carbohydrate esterases (CEs) and auxiliary activities (AAs). The database also comprises the non-catalytic carbohydrate-binding modules (CBMs), which are often found linked to degradative CAZymes, where their main function is to provide additional substrate-binding capabilities and improve overall enzyme efficiency [20].

Knowledge on CAZymes targeting the plant cell wall has mainly been generated from research on filamentous fungi and bacteria [21–23]. Various yeast species have been shown to grow on diverse and complex substrates, but their contribution to biomass degradation and as a source of CAZymes has been largely overlooked [21, 24]. Thus, yeast species represent an untapped source of CAZymes of potential industrial relevance. Moreover, yeasts capable of growing on diverse and complex substrates in challenging environments combined with a unicellular growth pattern, ease of cultivation and genetic manipulation make them attractive candidates as future biorefinery cell factories for consolidated bioprocessing (CBP) [25, 26].

Based on known CAZyme protein domains, it has recently become possible to annotate and predict CAZymes in whole genomes in a high-throughput manner using the automated online meta server dbCAN2 [27]. Moreover, advances in next-generation sequencing and bioinformatical tools have considerably increased knowledge of yeast genetics and evolution [28] and about a quarter of the approx. 1500 yeast species described to date have been sequenced [26, 29]. Together, these technical advances provide an opportunity to identify polysaccharide-degrading yeast species through bioinformatic mining, complementing time-consuming and labor-intensive bioprospecting approaches. The aim of this study was to identify polysaccharide-degrading yeasts by mining 332 yeast genomes from the Ascomycota phylum [26]. We used the results of the initial prediction of the species’ CAZyme repertoires to select a subgroup of CAZyme-rich yeasts for more in-depth characterization of polysaccharide metabolism and enzymatic activities. This bioinformatic-based approach allowed us to map phylogenetic clades rich in xylanolytic yeast species and identify additional highly xylanolytic non-sequenced yeast species.

## Methods

### Prediction of CAZymes by dbCAN2 in 332 ascomycetous yeasts

A bioinformatic analysis was carried out to identify CAZymes in the 332 ascomycetous yeasts [26]. Fasta files containing protein sequences were downloaded from Figshare (https://doi.org/10.6084/m9.figshare.5854692) in November 2019. The protein sequences in each fasta file were de-duplicated by clustering at 98% identity using CD-HIT [30] and cluster representatives were carried forward for further analysis. Hidden Markov Models (HMMs) for CAZymes were downloaded from dbCAN (http://bcb.unl.edu/dbCAN2/, version 8) [27]. Each sequence in the fasta files was matched against these HMMs using HMMER3 [31] with the -E flag set to filter hits with e-values below 10^–15^ as well as with the—domtblout flag to obtain an easily parsable output file. Hits covering less than 35% of the corresponding HMM model were removed. Additionally, if two domains showed more than 20% overlap on a single protein, only the domain with a better e-value score was retained. For each of the enzymes in the fasta files, potential signal peptides, indicating secretion, were also predicted using SignalP (http://www.cbs.dtu.dk/services/SignalP/) [32]. In these runs the -org flag was set to "euk" (for eukaryote), and the -format flag to "short" to obtain easily parsable output files. The final data was visualized, using the ETE toolkit, on phylogenetic trees comprising the 332 yeast species, likewise retrieved from the Figshare record (https://doi.org/10.6084/m9.figshare.5854692).

The entire bioinformatic analysis was carried out in an Anaconda environment (https://www.anaconda.com/; version 2018.12) using the Python programming language (version 3.7.1) on a Unix system. Python libraries used include Pandas version 0.25.1, re version 2.2.1, BioPython version 1.72 [33], ETE3 version 3.1.2 [34] and Matplotlib version: 3.3.2 [35]. Other dependencies include CD-HIT version 4.8.1, HMMER version 3.2.1, and SignalP version 5.0b.

### Yeast selection

Yeasts were selected based on their total number of predicted CAZymes and CAZyme functional activity clustering in polysaccharide degradation (Additional file 1: Table S1). In total, 40 sequenced yeasts and six non-sequenced yeasts were ordered from the ARS Culture Collection, USA (NRRL; https://nrrl.ncaur.usda.gov/). The selected sequenced species that we managed to cultivate in the lab are listed in Table 1. The six non-sequenced species were: *Sugiyamaella novakii* (CBS 8402), *Sugiyamaella smithiae* (CBS 5657), *Blastobotrys malaysiensis* (CBS 10336), *Blastobotrys illinoisensis* (CBS 10339), *Blastobotrys parvus* (CBS 6147) and *Scheffersomyces shehatae* (CBS 5813). All strains were either received freeze-dried in ampules which were re-grown in liquid yeast extract–peptone–dextrose (YPD) at room temperature, or as agar slants which were re-streaked on YPD agar plates and grown at room temperature. YPD contained 10 g L^−1^ yeast extract, 20 g L^−1^ peptone and 20 g L^−1^ glucose.

### Yeast growth characterization

Growth on polysaccharides was measured in both semi-solid and liquid media. Polysaccharides included wheat arabinoxylan (Megazyme, Ireland), birchwood glucuronoxylan (Sigma-Aldrich, Germany), xyloglucan (tamarind, Megazyme, Ireland), mixed-linkage β-1,3/1,4-glucan (barley, Megazyme, Ireland), galactomannan (guar/locust bean gum, Sigma-Aldrich, Germany), glucomannan (konjac, Sigma-Aldrich, Germany), curdlan (Merck, USA), poly-methylgalacturonan (Sigma-Aldrich, Germany), pectin (citrus, Sigma-Aldrich, Germany), carboxymethyl cellulose (Sigma-Aldrich, Germany), Avicel (Sigma-Aldrich, Germany) and potato starch (Sigma-Aldrich, Germany). For semi-solid growth, agar plates were prepared using autoclaved Delft minimal medium with different polysaccharides 0.2% (w/v) and 2% agar (w/v). The Delft media contained 5 g L^−1^ ammonium sulfate, 3 g L^−1^ potassium phosphate, 1 g L^−1^ magnesium sulfate, vitamins and trace metals as described previously [36], and pH was adjusted to 5 using 2 M KOH. Yeasts were inoculated in Delft medium 2% glucose (w/v) and grown at 30 °C, 150 rpm for 24 h before harvested, washed, and resuspended in water to a cell density of OD_600_ = 5.10 µl of the cell suspensions were spotted on plates that were then sealed with parafilm and kept at room temperature for 10 days before scoring growth. All strains were also spotted on agar plates either without any carbon source (where no strains were expected to grow) or with 2% glucose (where all strains were expected to grow). The *Saccharomyces cerevisiae* strain CEN.PK 113-7D that is unable to grow on polysaccharides was also included as a negative control. Growth was scored by visual inspection of colony thickness and size (including hyphae) in comparison to cell droplets on plates without carbon source. For growth in liquid cultures, yeasts were inoculated with a starting OD_600_ = 0.05 in Delft minimal media containing 10 g L^−1^ (w/v) of the different polysaccharides except curdlan, CMC and Avicel, and cultivated at 30 °C, 150 rpm for 72 h before determining growth through optical density (OD_600_) measurements. Yeast cultures that displayed optical densities of OD_600_ ≥ 0.2 were considered as growing on the respective polysaccharide.

To follow growth on xylan substrates over time, selected species were precultured at 30 °C, 150 rpm for 24 h in Delft medium containing 2% xylose (w/v). Here, xylose was selected as carbon source as it has previously been shown to induce expression of xylanases in other xylanolytic yeasts [24, 37]. Precultured cells were then inoculated in 250 µl Delft medium supplemented with 10 g L^−1^ xylan (either wheat AX or birchwood GX) to a starting OD_600_ = 0.2. While wheat AX was soluble in Delft medium, birchwood GX was not fully soluble. All yeast strains were grown in biological triplicates in a 96-well plate setup in a GrowthProfiler 960 (Enzyscreen, Netherlands). ‘Green Values’ (GV) measured by the GrowthProfiler correspond to growth based on pixel counts, and GV changes were recorded every 20 min for 72 h at 30 °C and 150 rpm.

### Xylanolytic activity determination

To quantify the xylanolytic yeasts’ secretome and cell-associated xylanase activities, the final cultures from the GrowthProfiler experiment were collected by centrifugation (2000×*g* 15 min) and xylanase activity was assayed in the cell-free supernatant or the intact cell pellets, respectively. The assay mixture consisted of a 175 µl xylan suspension of 10 g L^−1^ wheat AX or birchwood GX and 50 mM sodium acetate buffer (pH 5.5) added to cell pellets or 25 µl cell-free supernatants mixed in a 96-well plate. The mixture was incubated at 30 °C for 30 min followed by immediate chilling on ice. Reducing sugar ends released by xylanases was determined by the dinitrosalicylic acid (DNS) method [38] as end point assay. All enzymatic measurements were performed in triplicates. One unit of enzyme activity was defined as the amount of enzyme required to release 1 µmol of reducing sugars in 1 min under the assay conditions. Volumetric activity (U mL^−1^) was calculated by converting mM reduced sugar to Units by multiplying with total assay volume (L), dividing with assay time (min) and then dividing with sample volume (L) as described previously [39].

### Phylogenetic analysis

Phylogenetic trees of GH10 and 11 xylanases were constructed using the identified yeast enzymes as well as sequences from 259 characterized GH10 members and 208 characterized GH11 members retrieved from the CAZy database (www.cazy.org), respectively. The sequences were aligned using MUSCLE (https://www.ebi.ac.uk/Tools/msa/) [40], and then submitted for tree building using the online Iqtree tool http://iqtree.cibiv.univie.ac.at/ with 1000 bootstrap alignments and viewed in MEGA-X as Newick trees [41]. For species phylogenetic analysis, Internal Transcribed Spacer (ITS) nucleotide sequences from xylanolytic yeasts, their closely related species and *Schizosaccharomyces pombe* as outgroup were aligned using ClustalW, and a maximum likelihood (ML) phylogenetic tree with bootstrap value 1000 was constructed using MEGA-X.

### In-gel proteomics of the GH11 enzyme from *Blastobotrys mokoenaii*

Supernatants from yeast cultures of *Blastobotrys mokoenaii* grown in Delft minimal medium with 10 g L^−1^ birchwood GX or wheat AX (72 h, 30 °C, 150 rpm) were concentrated using 10 kDa ultra centrifugal filters (Amicon, Merck, Germany) by centrifugation (2000×*g*, 10 min, repeated 3×). Secreted proteins were identified by sodium dodecyl sulphate–polyacrylamide gel electrophoresis (SDS-PAGE). A protein at ~ 24 kDa was cut out from the gel using a scalpel and kept at − 20 °C before sent for proteomic analysis. The protein identity was confirmed by MS/MS analysis as a 23.19-kDa GH11 xylanase with the following predicted protein sequence (217 residues):

MKLSNAITAICAAAVLAAPLEEEEVAKRSVTPSSTGTNNGYYYSFWSDGGGDVTYTNGNGGSYSVEWTNCGNFVGGKGWNPGAAREINFSGSFNPSGNGYLSVYGWTTNPLVEYYIVESYGDYNPGTAGTFLGTVDSDGSTYDIYKAVRTNAPSIEGTATFDQYWSIRRNHRTSGTVNTGNHFNAWAQHGLQLGTHNYQIVATEGYQSSGSSSITVS.

## Results

### CAZyme abundance and distribution in ascomycetous yeasts

To identify polysaccharide-degrading yeasts, the dbCAN2 meta server was used to scan the genomes of 332 yeast species within the Ascomycota phylum to predict and compile their encoded CAZymes [26, 27]. It is important to note that the 332 genomes included in the dataset are not all complete [26], and therefore, the list of CAZymes is likely incomplete. Nonetheless, we identified a total 217 different CAZyme families, with GT and GH as the most predominant classes. The full genetic CAZyme prediction and associated protein sequences can be downloaded from Zenodo (https://zenodo.org/record/4548336/export/hx#.YLSzGqgzaUk, https://doi.org/10.5281/zenodo.4548335).

**Fig. 1:**
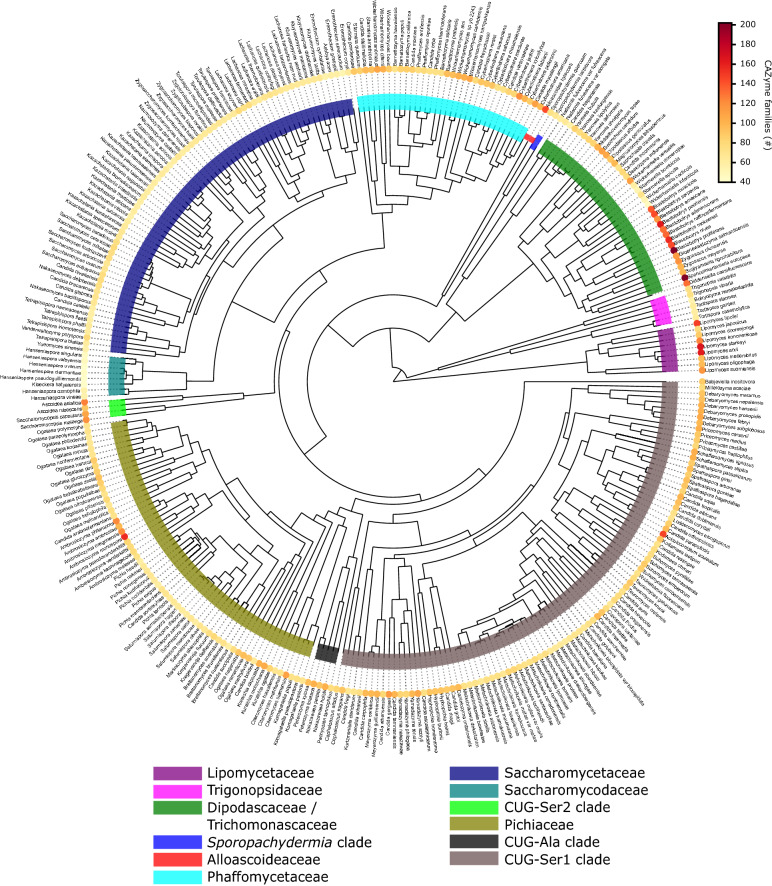
CAZyme abundance in 332 budding yeasts. The total number of predicted CAZymes (GTs excluded) in each yeast species is represented by a heat signature ranging from light yellow to dark red with increasing numbers of predicted CAZymes

The 332 yeasts encoded on average 152 CAZymes with 20 yeasts having more than 200 CAZymes. Yeasts containing the highest number of CAZymes primarily belong to the Trichomonascaceae clade, followed by species in the Lipomycetaceae and the Pichiaceae clades. Species with low amounts of CAZymes include *Hanseniaspora* and *Eremothecium* species. With our focus being on potential polysaccharide-degrading yeasts and their respective CAZymes, GTs involved in biosynthesis of disaccharides were not included in subsequent analyses. An overview of the abundance of CAZymes (except GTs) in individual yeast species throughout the phylogenetic tree can be viewed in Fig. 1, with increasing CAZyme numbers represented by yellow to dark red color. From this initial analysis, we can conclude that some clades more than others appear to be hotspots for identification and characterization of yeast CAZymes.

### CAZyme annotation in putative polysaccharide-degrading yeasts

**Fig. 2:**
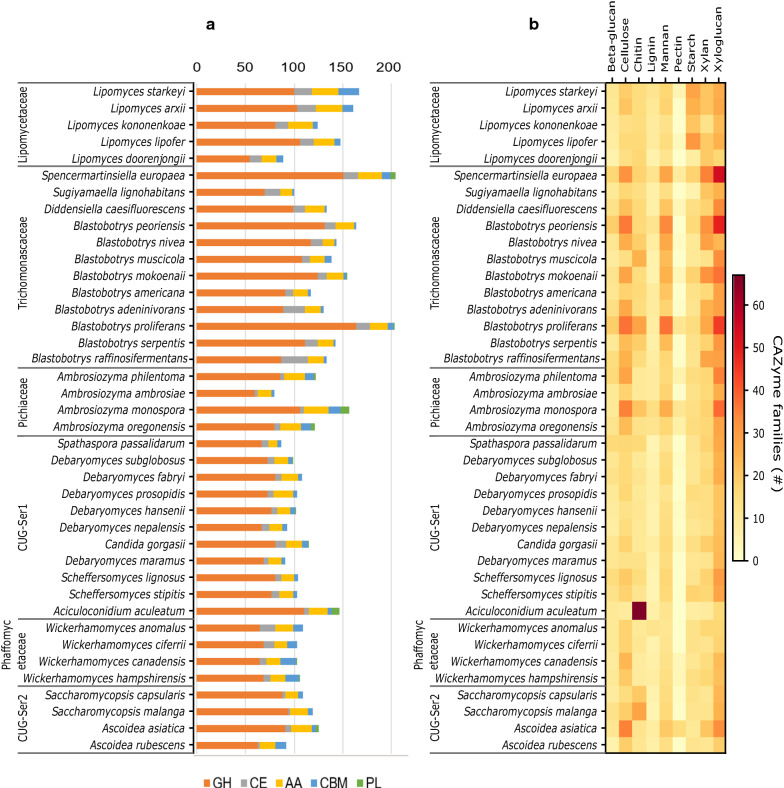
Total number of CAZymes (except GTs) in the 40 selected yeasts and their grouping by function. **a** Total number of CAZymes in each selected species. **b** CAZyme families from the same species grouped by predicted function in polysaccharide degradation. Dark red and red-colored squares indicate high number (#) of CAZymes with predicted activity towards the listed polysaccharide. Please note that the heatmap is depicting the total number of CAZyme-encoding genes belonging to families known to degrade specific polysaccharides, and thus heat signatures from polysaccharides with very few CAZymes needed for depolymerization (e.g., β-glucan) may be skewed compared to more complex polysaccharides (such as xylan) requiring many CAZymes. Poly-specific enzyme families such as GH5 and GH3 may also show false positive activities as their members have shown activities on several different β-1,4-linked glycans, e.g., xylanase, mannanase, glucanase, glucosidase, galactanase [19]. GH5 enzymes were assigned to cellulose, mannan, xylan, and xyloglucan, while GH3 were assigned to β-glucan, cellulose, xylan and xyloglucan. CBM, carbohydrate-binding module; CE, carbohydrate esterase; GH, glycoside hydrolases; PL, polysaccharide lyase

To relate and confirm genetic CAZyme predictions to real capacity of polysaccharide utilization, 40 yeasts from six different phylogenetic clades were selected for further characterization. The species were chosen based on their total number of predicted CAZymes and the clustering of their CAZymes by functional activity involved in polysaccharide degradation. The distribution of the different enzyme classes (excluding GTs) and CBMs in the selected yeasts is shown in Fig. 2a. The GHs showed the highest variation in number among species while relatively few CBM and PL families were predicted. The yeast with highest number of CAZymes (excluding GTs) was *Spencermartinsiella europaea* with 204 predicted CAZymes followed by *Blastobotrys proliferans* (203) from the same clade, then *Lipomyces starkeyi* (167) from the Lipomycetaceae clade. These numbers are around twice the number of CAZymes found in the more commonly studied yeasts such as *Saccharomyces cerevisiae* (79) and *Schizosaccharomyces pombe* [22]. Notably, another five *Blastobotrys* species from the Trichomonascaceae clade also ranked among the top 25 yeasts in terms of absolute CAZyme numbers. In addition, we grouped the yeasts’ CAZymes by predicting functional polysaccharide degradation activities (Additional file 1: Table S1), e.g., β-glucanases, cellulases, chitinases, lignin-degrading enzymes, mannanases, pectinases, starch degrading enzymes, xylanases and xyloglucanases [18] and created a heatmap based on the resulting number of enzymes (Fig. 2b). The analysis suggests that the yeasts from the Trichomonascaceae clade have diverse enzyme portfolios and with a particular enrichment of mannan-, xylan-, xyloglucan-, and cellulose-degrading CAZymes. The Lipomycetaceae clade appears rich in starch degrading CAZymes, while *Aciculoconidium aculeatum* in the CUG-Ser1 clade contains multiple enzymes for chitin degradation with a total of 57 predicted GH18 chitinases. In general, relatively few CAZymes associated with pectin and lignin degradation were predicted in the ascomycetous yeast genomes (Fig. 2b). Collectively, the results suggest that the assessed yeasts are equipped with a range of different polysaccharide-degrading enzymes, where some species seem specialized to degrade specific polysaccharides while others appears to be polysaccharide generalists.

### Growth characterization on different polysaccharides

To determine if polysaccharides could support growth for the 40 selected ascomycetous yeasts, the yeasts were cultivated on agar plates with semisolid minimal media (Delft) supplemented with different polysaccharides as the sole carbon source. Growth on xylan, xyloglucan, β-glucan, galactomannan, glucomannan, pectin and poly-methylgalacturonan polysaccharides was also confirmed in liquid cultures and the accumulated growth results are shown in Table 1. Several species—*Lipomyces doorenjongii, Lipomyces kononenkoae, Lipomyces lipofer, Lipomyces starkeyi, Aciculoconidium aculeatum, Ambrosiozyma ambrosiae, Ascoidea rubescens* and *Blastobotrys nivea*—did not grow in the pre-cultures and were therefore discarded from further analysis*.* In general, growth on agar plates corresponded well with the increased optical density (OD_600_ > 0.2) observed in liquid cultures, though some species from the CUG-Ser1 clade, particularly *Scheffersomyces* species, showed better growth in liquid culture than on agar plates with mannan-based, pectin and xyloglucan polysaccharides (Table 1). In accordance with the CAZyme heatmap (Fig. 2b), species from the Trichomonascaceae clade showed substantial growth on hemicellulosic substrates, particularly xylans, β-glucan, glucomannan and galactomannan. Also yeasts from the CUG-Ser1 and Phaffomycetaceae clades showed growth on xylan, whereas those from the Pichiaceae clade did not. Some of the herein characterized species have been identified as xylan-growers also in other screens, for example *Scheffersomyces stipitis, Sugiyamaella lignohabitans* and *Spencermartinsiella* sp. [42] while, to the best of our knowledge, other species such as *Blastobotrys serpentis, Blastobotrys peoriensis* and *Scheffersomyces lignosus* have so far escaped attention in this regard [43, 44].

**Table 1:**
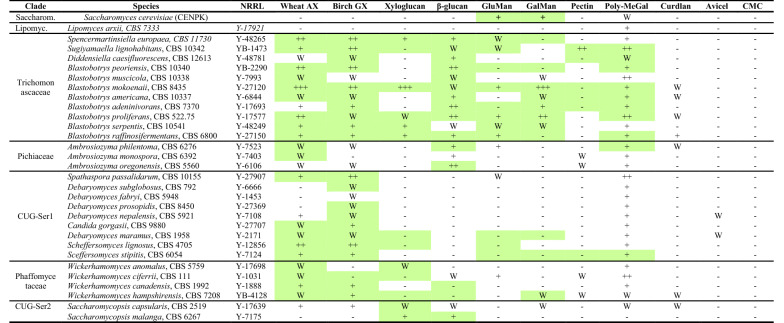
Overview of budding yeast growth assessment on agar plates and liquid cultures using different polysaccharides

Growth was scored by visual comparison to a negative control plate not containing a carbon source and by the difference in colony thickness and size (including hyphae, if present)

Growth was ranked from + to +++, where + was regular growth and +++ extensive growth, while W indicates weak growth and − no growth. Growth after 72 h in liquid cultures > OD = 0.2 is indicated by a green color

AX, arabinoxylan; GX, glucuronoxylan; GluMan, glucomannan; GalMan, galactomannan; Poly-MeGal, poly-methylgalacturonan; CMC, carboxymethyl cellulose; Saccharom., Saccharomycetaceae; Lipomyc., Lipomycetaceae

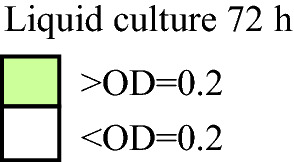

In opposite to hemicellulosic substrates, the assessed yeasts did not grow well on cellulose despite predictions of cellulase activities (Table 1, Fig. 2b). In line with these results, a large-scale screen to identify wild cellulolytic yeasts showed that only 16 of 390 strains grew on cellulose and just 5 had significant enzyme activity levels [45], indicating that most yeasts are unable to utilize crystalline cellulose [24]. Overall, we can conclude that the polysaccharide-degrading ascomycetous yeasts identified in this study display better growth on hemicellulosic substrates compared to cellulosic substrates in accordance with previous studies.

### Growth and enzymatic activities of xylan-utilizing yeasts

**Fig. 3:**
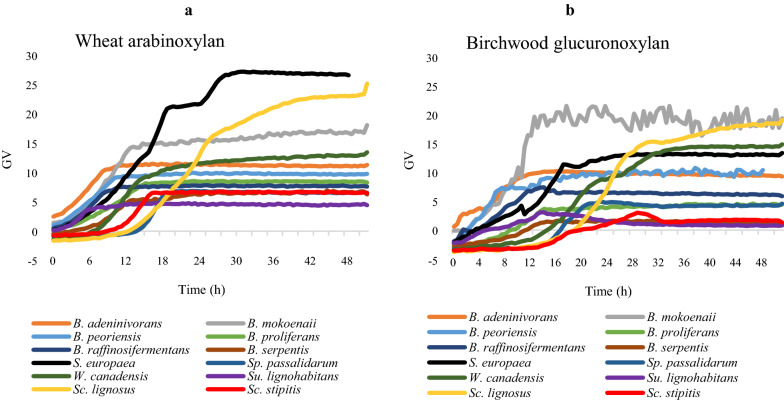
Growth profiles of 12 xylanolytic yeasts in Delft minimal medium containing 10 g/L of either **a** wheat arabinoxylan or **b** birchwood glucuronoxylan. GV = Green Value (corresponding to growth based on pixel counts, as determined by a GrowthProfiler instrument). Growth profiles are shown as averages of triplicates

To further characterize the top xylan-utilizing yeast species, we determined their growth profiles over time in both wheat AX and birchwood GX (Fig. 3). The xylanolytic yeasts showed different growth profiles and reached stationary phase between 8–28 h, with some species showing biphasic growth curves. Notably, *B. mokoenaii, Sp. europaea, Sc. lignosus* and *Wickerhamomyces canadensis* reached the highest optical densities in both xylans, although the yeasts’ growth profiles differed somewhat between the two substrates (Fig. 3a, b).

**Fig. 4:**
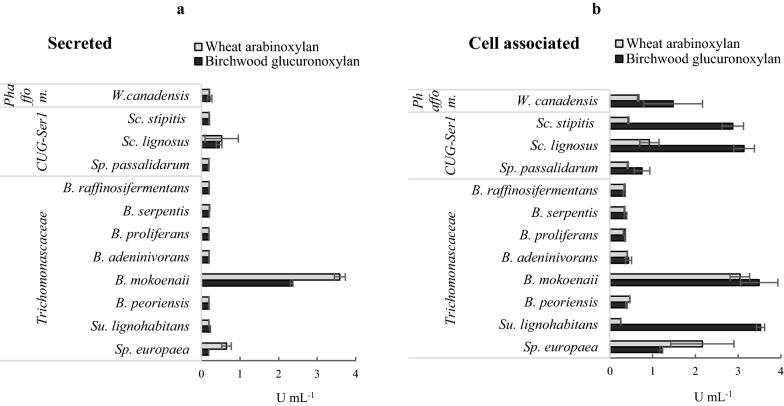
Xylanolytic yeast activities in liquid cultures. Volumetric activities of **a** secretome xylanases and **b** yeast cell-associated xylanases in wheat arabinoxylan (grey) and birchwood glucuronoxylan (black) determined at 30 °C after growth on xylan in liquid medium for 72 h. Phaffom. = Phaffomycetaceae clade

Next, the xylanolytic yeasts were characterized in terms of xylanase activities. Both the secretome and cell-associated enzymatic activities were assayed to gain deeper insight into the xylanolytic strategies used by these species. Xylanase activity of the secretome was particularly high in *B. mokoenaii* for both types of xylans, with a higher activity on wheat AX compared to birchwood GX (3.6 and 2.3 U mL^−1^, respectively) (Fig. 4a). These values were 7.2-fold higher than those of *Sc. lignosus* that had the second highest secretome activity values, and also higher than what has been reported previously on yeasts that secrete xylanases [37]. This indicates that *B. mokoenaii* possesses a unique xylanolytic strategy among the studied species. *B. mokoenaii* also had a high cell-associated xylanase activity on both wheat AX and birchwood GX, a feature shared with several other species. These included the other top xylan-growing species *Sp. europaea, Sc. lignosus*, and *W. canadensis* (Figs. 3 and 4b), which all showed good correlation between enzyme activity and growth. However, for several other yeasts, the correlation between measured xylanase activity and growth characteristics was ambiguous. For example, yeasts such as *B. adeninivorans* and *B. peoriensis* with intermediate growth in both xylans showed only modest xylanolytic activities (0.2–0.3 U mL^−1^), whereas *Sc. stipitis* and *Su. lignohabitans* showed high xylanase activities (0.4–2.8 U mL^−1^) but only moderate xylan growth (Figs. 3, 4). Overall, the diverse profiles in terms of growth, enzymatic activities and xylan substrate preferences point towards different yeast strategies for degradation and utilization of xylan.

### CAZyme analysis in xylanolytic yeast species

To connect the experimentally measured xylanolytic activities with the predicted CAZymes, we identified all putative xylanolytic CAZymes for each of the top 12 xylan-growing yeasts (Table 2). Overall, the yeasts, coming from three clades, have similar numbers of genes encoding CEs with expected roles in de-acylation of polysaccharides, and GH3 enzymes predicted to act as exo-β-glycosidases on oligosaccharides. The species from the Trichomonascaceae clade have a more diverse and abundant xylanolytic CAZyme distribution compared to yeasts from other clades. The top-performing xylanolytic yeast *B. mokoenaii* encodes a putative GH11 xylanase, which is a unique trait within the whole 332 yeast dataset. We were able to detect the GH11 protein with a molecular size of 23.19 kDa in the secretome of *B. mokoenaii* grown in medium containing wheat AX or birchwood GX, using in-gel proteomic MS/MS analysis (Additional file 2: Fig. S1). The GH11 gene can be found in the genome position 29298–29948 in the GenBank sequence ID: PPJM02000065.1. *B. mokoenaii* is also unique in that it possesses two gene copies for GH5 enzymes from subfamily 7 (GH5_7; putative endo-β-1,4-mannanases) and a GH62 α-l-arabinofuranosidase. Further, *B. mokoenaii* encodes a GH30_7 enzyme (putative exo-β-1,4-xylanase or glucuronoxylanase) in common with only two other yeasts that also scored high in our assays: *Su. lignohabitans* and *Sp. europaea*. Indeed, all eight species in the Trichomonascaceae clade have predicted GH30 enzymes and some species have putative GH67 α-glucuronidases as well as GH43 and GH51 enzymes predicted to be α-l-arabinofuranosidases), indicating abilities to target complex GAX. A similar setup is not found in the CUG-Ser1 and Phaffomycetaceae clades. However, the CUG-Ser1 clade species possess a putative GH115 α-glucuronidase, potentially enabling them to hydrolyze glucuronic acid side chains present in birchwood GX.

**Table 2:** Xylanolytic CAZyme predicted from whole-genome sequenced xylanolytic yeasts

Clade	Species	CE (332/332)	GH3 (263/332)	GH5 (324/332)	GH10(5/332)	GH11 (1/332)	GH30 (11/332)	GH43 (22/332)	GH51 (39/332)	GH62 (1/332)	GH67 (4/332)	GH115 (28/332)	Total
Trichomonascaceae	*Sp. europaea*	CE1, CE4, CE15	GH3(11)	GH5_5(2), GH5_9(2), GH5_12(2), GH5_22(4), GH5_49	GH10(2)		GH30_5, GH30_7	GH43_14, GH43_24	GH51		GH67	GH115(3)	36
	*Su. lignohabitans*	CE1(2), CE4(3), CE15	GH3(5)	GH5_9(2), GH5_12(2), GH5_22(2), GH5_49	GH10(2)		GH30_7		GH51			GH115	23
	*B. peoriensis*	CE1, CE4	GH3(15)	GH5_9(3), GH5_12(2), GH5_22(5), GH5_49	GH10		GH30_3(3)		GH51				33
	*B. mokoenaii*	CE1, CE4	GH3(8)	GH5, GH5_5, **GH5_7**(2), GH5_9(2), GH5_12, GH5_22(2), GH5_49		**GH11**	GH30_5, GH30_7	GH43_6, GH43_24	GH51(3)	**GH62**	GH67	GH115(2)	32
	*B. adeninivorans*	CE1, CE4(2)	GH3(8)	GH5_9(3), GH5_12(2), GH5_44, GH5_47			GH30_3	GH43_6	GH51		GH67		22
	*B. proliferans*	CE1(2), CE4	GH3(12)	GH5_5(3), GH5_9(2), GH5_12, GH5_31, GH5_49			GH30_3		GH51(3)				27
	*B. serpentis*	CE1, CE4	GH3(5)	GH5, GH5_9(3), GH5_12, GH5_22, GH5_49			GH30_3(2)		GH51(2)				18
	*B. raffinosifermentans*	CE1, CE4(2), CE5(6)	GH3(9)	GH5_9(3), GH5_12(2), GH5_49			GH30_3	GH43_6	GH51		GH67		27
CUG-Ser1	*Spa. passalidarum*	CE1, CE4(2)	GH3(9)	GH5, GH5_9(3), GH5_22, GH5_49								GH115	19
	*Sc. lignosus*	CE1, CE4,	GH3(7)	GH5_5, GH5_9(3), GH5_12, GH5_22(2), GH5_49	GH10(2)							GH115	20
	*Sc. stipitis*	CE1, CE4,	GH3(7)	GH5_9(2), GH5_12, GH5_22 (3), GH5_49	GH10							GH115	17
Phaff.	*W. canadensis*	CE1(3), CE4	GH3(5)	GH5_9(2), GH5_12, GH5_22, GH5_49									14

CAZyme families marked in bold are unique enzymes to the species within the 332-yeast dataset. Total number of species containing each CAZyme family is marked by (x/332). Copy number of each enzyme is stated in parenthesis next to the enzyme

*CE* carbohydrate esterase, *GH* glycoside hydrolase, *Phaff.* Phaffomycetace

Species displaying good xylanolytic activity (2–4 U mL^−1^) almost all possess predicted GH10 (*Sp. europaea*, *Su. lignohabitans*, *Sc. stipitis* and *Sc. lignosus)* or GH11 (*B. mokoenaii*) xylanases (Fig. 4, Table 2). An interesting exception is *W. canadensis*, which does not appear to encode either GH11, GH10 or GH30 xylanases. However, it possesses putative GH5_9, GH5_22 and GH5_49 CAZymes in common with most of the xylanolytic species listed in Table 2, suggesting that some of these CAZymes may be novel xylanases. No xylanase activities have yet been confirmed in the mentioned GH5 subfamilies, and in fact no GH5_49 enzymes have to date been biochemically characterized [18]. Although we cannot completely rule out that the lack of genes encoding known xylanases in *W. canadensis* is due to an incomplete genome assembly, this species and its putative xylanases deserve further characterization outside the scope of this study.

### Phylogenetic analysis of GH10 and GH11 xylanases

**Fig. 5:**
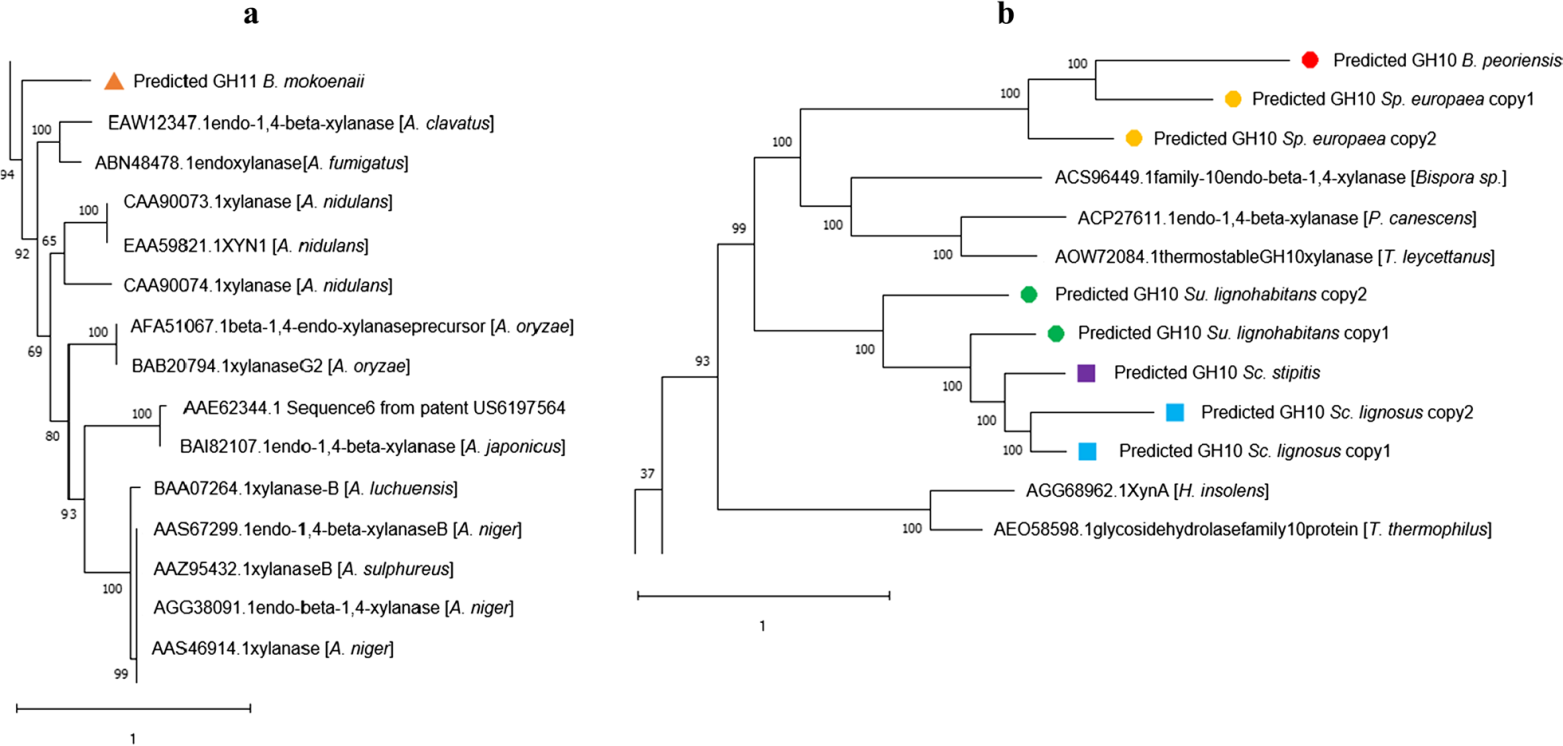
Phylogenetic analysis of GH11 and GH10. **a** Phylogenetic placement of the GH11 xylanase from *B. mokoenai*i (orange triangle) and **b** Phylogenetic placement of the GH10 xylanases from *Sp. europaea* (yellow circles), *Su. lignohabitans* (green circles), *B. peoriensis* (red circle), *Sc. lignosus* (blue squares) and *Sc. stipitis* (purple square). The molecular phylogenetic analysis was performed using full protein sequences from 259 GH10 and 208 GH11 characterized enzymes using Newick tree model from MUSCLE alignment with 1000 boot strap replicates. The numbers at each branch indicate bootstrap values and tree topology confidence. Trees are drawn to scale with branch lengths measured in numbers of substitutions per site. Scale bars represent 1.0 substitutions per nucleotide position

To investigate the origin of the genes encoding the identified GH10 and GH11 members in the ascomycetous yeasts, we determined the phylogenetic relationships of these enzymes with 259 characterized enzymes from GH10 and 208 from GH11, listed in the CAZy database [18]. Phylogenetic trees displaying all characterized enzymes can be viewed in Additional file 3: Fig. S2. The GH11 xylanase from *B. mokoenaii* shows the highest sequence identify (71.95%) to the xlnB xylanase from *Aspergillus nidulans* FGSCA4 with confirmed ability to hydrolyze oat-spelt xylan [46], suggesting a similar function of the putative *B. mokoenaii* enzyme (Fig. 5a). All GH10 copies from *Sp. europaea, Su. lignohabitans, B. peoriensis, Sc. lignosus and Sc. stipitis* clustered to the same branch of the phylogenetic tree, together with characterized xylanases from the filamentous fungi *Talaromyces leycettanus*, *Penicillium canescens* and *Bispora sp.MEY-1* (Fig. 5b). Thus, we can conclude that all yeast GH10 and GH11 are of Ascomycota origin and likely have xylanolytic activity. The presence of these genes in the yeast genomes could be a result of horizontal gene transfer within the phylum, or that these genes have been specifically retained by a small number of (ancestral) yeast species after the split between Pezizomycotina (filamentous fungi) and Saccharomycotina (yeasts) in the Ascomycota phylum. In favor for the gene retention explanation model, Morel and co-authors have shown that the genome of the yeast *Geotrichum candidum* within the Trichomonascaceae clade contains a few hundred genes that are orthologous to predicted genes in filamentous fungi rather than other sequenced Saccharomycotina yeasts [47]. Moreover, *B. mokoenaii* possesses several other unique CAZymes, including ones from GH62 and GH12 that also show high sequence identity to CAZymes from *Aspergillus* species (77% to *A. pseudonomiae* and 62% to *A. flavus*, respectively), further supporting this model.

### Identification of novel xylanolytic species by phylogenetic association

**Fig. 6:**
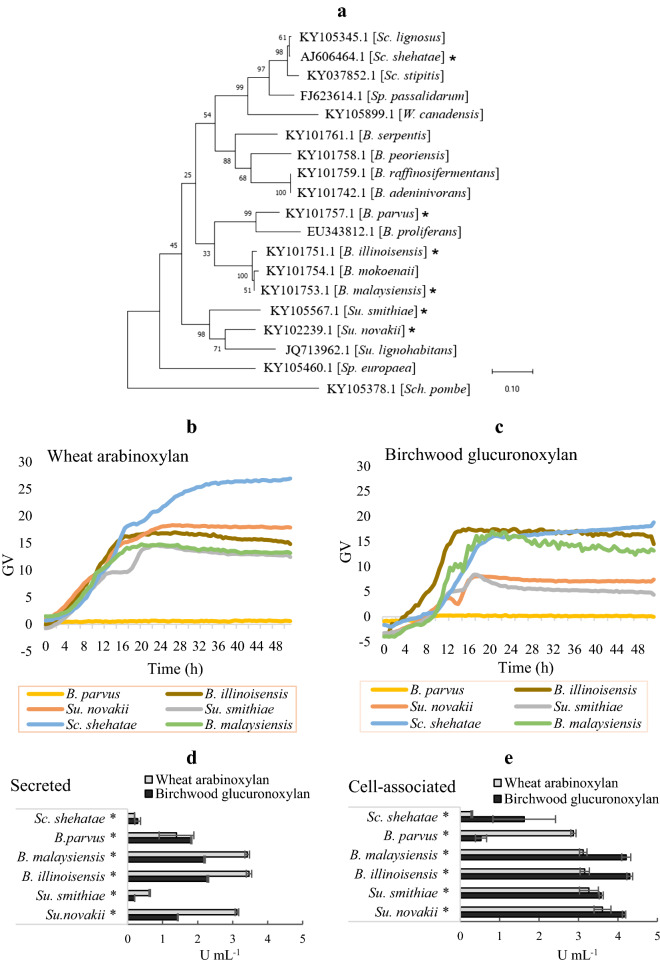
Characterization of non-sequenced xylanolytic yeasts. **a** Phylogenetic analysis of 19 *Blastobotrys*, *Sugiyamaella and Scheffersomyces* species as well as *Schizosaccharomyces pombe* serving as outgroup. The molecular phylogenetic analysis was based on ITS sequences using maximum-likelihood model from ClustalW alignment with 1000 bootstrap replicates. The numbers at each branch indicate bootstrap values and tree topology confidence. The tree is drawn to scale, with branch lengths measured in the number (0.2) of substitutions per site. Growth profiles of xylanolytic yeasts grown in Delft medium containing 10 g L^−1^ of **b** wheat arabinoxylan and **c** birchwood glucuronoxylan. Yeasts were grown for 48 h at 30 °C. GV = Green Value (corresponding to growth based on pixel counts, as determined by a GrowthProfiler instrument). **d** Secretome and **e** cell-associated volumetric xylanase activities on wheat arabinoxylan (grey) and birchwood glucuronoxylan (black) determined at 30 °C after growth in xylan-containing liquid medium for 72 h. Stars (*) symbolizes non-sequenced species

The successful approach of using CAZyme prediction to identify xylan-degrading yeasts in CAZyme-rich clades, prompted us to scout for additional, novel xylanolytic species through phylogenetic association. Six non-sequenced yeast species phylogenetically closely related to the highest scoring xylanolytic species found in this study were therefore included in another round of characterization; *Sugiyamaella novakii* (CBS 8402), *Sugiyamaella smithiae* (CBS 5657), *Blastobotrys malaysiensis* (CBS 10,336), *Blastobotrys illinoisensis* (CBS 10,339), *Blastobotrys parvus* (CBS 6147) and *Scheffersomyces shehatae* (CBS 5813) (Fig. 6a). All species except *Sc. shehatae* have so far largely escaped scientific attention, and genomic information except for ITS and ribosomal RNA sequences is almost completely missing [37, 42, 44, 48]. The yeast growth profiles in Delft minimal media containing wheat AX and birchwood GX as carbon sources and the secretome and cell-associated xylanolytic activities and can be seen in Fig. 6b-e. All six species showed secreted or cell-associated xylanolytic activities, or both, and all except *B. parvus* grew on both xylan substrates. This species, however, displayed high xylanolytic activity (1.8–2.8 U mL^−1^) on both xylan types (Fig. 6b-e). Interestingly, *Sc. shehatae* reached the highest green values (26.7 GV) in wheat AX out of all the xylanolytic yeasts characterized in this study (Fig. 6b) and *Su. smithiae* and *Su. novakii* showed high activity on both xylans (in contrast to *Su. lignohabitans* which seem to prefer birchwood GX) (Figs. 4, 6d, e). Overall, these results show that CAZyme-rich clades are treasure troves for identifying xylanolytic yeast species, and sequencing and characterization of the new yeasts will most likely lead to additional discoveries of CAZymes with potential industrial value.

## Discussion

Complete depolymerization of complex lignocellulosic polysaccharides requires a repertoire of enzymes that act together on the different chemical bonds [14]. While CAZyme systems from filamentous fungi and bacteria have been studied for decades, yeast species have received considerably less attention. However, since yeasts are key industrial workhorses, elucidating their plant cell wall-degrading potential may be of great benefit for the development of efficient CBP strains able to both produce the enzymes needed for biomass degradation and convert the released sugars into valuable products [49]. Non-conventional, xylanolytic yeasts can potentially be developed into future CBP cell factories. Alternatively, the strategies these yeasts use may be directly transferrable to industrial *Saccharomyces* species in a manner that is not feasible for systems used by filamentous fungi or bacteria. We here present a strategy of high-throughput mining of genomes for putative CAZymes followed by growth studies and enzymatic investigation, through which we identified several novel yeast species displaying high xylanolytic activities and seemingly diverse strategies for hemicellulose utilization.

### Correlation between predicted CAZymes, growth and enzymatic activities

Identification of CAZyme-encoding genes in a genome is a promising lead in finding polysaccharide-utilizing species, but it does not per say reveal whether the genes are actually expressed into functional enzymes by the organism. Moreover, a series of enzymatic activities is required to degrade complex polysaccharides into oligo- and monosaccharides that can be taken up and metabolized by the microorganism. To find yeasts equipped with all enzymes needed to hydrolyze and grow on plant polysaccharides, we initially selected CAZyme-rich yeasts rather than cherry-picked species containing single GHs of interest. By doing so, our hope was also to increase the chances of capturing species equipped with CAZymes from poly-specific GH families or even GH families not yet associated with the targeted polysaccharides. Overall, this proved to be a highly successful approach, as we identified multiple polysaccharide-degrading species not captured through previous bioprospecting campaigns [37, 42, 48].

Among the CAZyme-rich species characterized in this study, we identified several yeast species from the Trichomonascaceae*,* CUG-Ser1 and the Phaffomycetaceae clades that were able to grow on xylan substrates. However, not all species enriched in CAZymes associated with xylan degradation grew according to the CAZyme-predictions and some surprisingly displayed relatively high xylanolytic activities but only modest xylan-growth, suggesting an inability to utilize the released oligosaccharides. Moreover, the CAZyme heatmap indicated potential for cellulolytic activity in several species, but this was not observed in the growth studies for the soluble cellulose analog CMC or crystalline and insoluble Avicel. For efficient degradation of cellulose to glucose, endoglucanase, cellobiohydrolase, lytic polysaccharide monooxygenase and β-glucosidase activities are generally regarded as needed [50–53]. The inability of yeasts to grow on cellulose may be due to the absence of one or several of the enzyme families known to encode such enzymes, such as GH6 (totally absent in the 332 yeasts) and GH7 (only present in *B. peoriensis*) and GH12 (only found in *B. mokoenaii*). These results are well correlated with the lack of literature describing cellulose-growing yeasts, and only a few studies have reported cellulolytic enzymatic activities in yeasts [45, 55, 56]. Further analysis is needed to decipher the relationships between bioinformatic CAZyme prediction and measured growth and enzymatic activities, but our results clearly emphasize the need to confirm bioinformatic predictions with wet lab characterization.

### Xylanolytic strategies in ascomycetous yeasts clades

The xylanolytic yeasts identified in this study display volumetric xylanase activities that range between 0.9 and 4.3U mL^−1^ (cell-associated and secreted activities combined), which can be compared to the xylanolytic activities of 28–30 U mL^−1^ reported for filamentous fungi *Aspergillus niger, Aspergillus flavus and Trichoderma viride* [57, 58] and also to previous reports of *Sc. stipitis* (1.6 U mL^−1^) and *Su. lignohabitans* (0.29 U mL^−1^) [48, 59]. Strikingly, the different yeast species characterized here seem to have versatile approaches to degrade xylan. Most of the species that showed high enzymatic activities also possess CAZymes from the well-studied xylan-associated GH families GH5, GH10, GH11 and GH30. GH10 members were found in species in the Trichomonascaceae and CUG-Ser1 clades that preferentially display cell-associated xylanolytic activities. In contrast, *B. mokoenaii* was the only species identified with a GH11 enzyme, which seems to be secreted in a soluble form, i.e. not attached to the yeast cell surface. In addition to GH10 and GH11, species in the Trichomonascaceae clade encoded enzymes from a range of additional families potentially containing xylan-acting enzymes that are completely lacking in the other clades. For example, enzymes from subfamily GH30_5 (putative endo-β-1,6-galactanase) were present in *B. mokoenaii* and *Sp. europaea,* while a putative exo-β-1,4-xylanase or glucuronoxylanase from GH30_7 was present in *B. mokoenaii, Su. lignohabitans* and *Sp. europaea* – three of the top scoring xylanolytic species found in our study. Yeasts in this clade were also found to contain genes for different xylanolytic de-branching enzymes, e.g., likely GH43, GH51 and GH62 α-l-arabinofuranosidases and GH67 α-glucuronidases. These could enable de-branching of complex xylans and reduce steric hindrances thus promoting the access to the xylan backbone for main-chain cleaving xylanases [60].

Along with the Trichomonascaceae species, we also identified xylanolytic species such as *W. canadensis* where the expected xylan-depolymerizing CAZyme setup seems to be absent. As mentioned above, the lack of predicted xylanolytic CAZymes in *W. canadensis* could be due to an incomplete genomic sequence [26]. However, other xylanolytic yeasts such as *Sp. passalidarum* and *Candida intermedia* [37, 42] for which complete or almost complete genomes exist, obvious xylanolytic enzyme-candidates are also missing (this study and unpublished results). This supports the hypothesis that these yeasts possess novel xylanases that should be explored more thoroughly. Further studies in terms of gene deletion studies and/or heterologous expression and characterization of individual enzymes are needed to conclusively assign different enzyme activities of these putative enzymes.

### Future outlook

We here present the identification of several novel ascomycetous yeast species that can grow on polysaccharides and particularly on xylans. Future research includes careful physiological characterization of select species from the Trichomonascaceae, CUG-Ser1 and Phaffomycetaceae clades, to determine their precise growth requirements and full substrate ranges, tolerance-levels to industrial stressors and product portfolios. Additionally, xylanases are in high industrial demand for production of textiles, pulp and paper as well as in modern biotechnology for production of, for example, functional foods and feeds. Characterization of the many putative CAZymes identified in yeasts may provide new enzyme features in terms of fold, stability and specificity with potential to improve current processes and enable new applications. To assign physiological roles and substrate specificities to these enzymes, heterologous expression, purification and biochemical characterization will be needed.

## Conclusions

Yeast biodiversity presents a huge, untapped resource for present and future industrial applications such as CBP in terms of desirable microbial phenotypes and novel CAZyme discovery. In this study, we have developed a bioinformatic pipeline to rapidly process and predict CAZymes in a large number of genome sequenced ascomycetous yeasts. The resulting CAZyme predictions combined with growth and enzymatic activities assays enabled identification of several novel xylanolytic yeasts. Moreover, additional non-sequenced species with xylan-degrading capacity were identified through phylogenetic association. Many species identified and characterized here show equal or better xylanolytic activities compared to described species in literature such as *Scheffersomyces* and *Sugiyamaella* species, highlighting the potential of the approach. Collectively, the results presented expand our current knowledge on polysaccharide-degrading ascomycetous yeasts and opens up for numerous follow-up studies on yeast physiology and CAZyme characterization. The knowledge generated through such studies will be of high importance for the optimization of lignocellulosic biomass conversion processes.

## Supplementary Information

**Additional file 1: Table S1.** Table S1. CAZyme families grouped by polysaccharide degradation function for heatmap generation.

**Additional file 2: Fig. S1.** SDS-PAGE gel showing proteins secreted by B. mokoenaii after three days of growth in xylan containing Delft medium.

**Additional file 3: Fig. S2.** Phylogenetic analysis of GH11 and GH10 xylanases.

## Data Availability

All data generated and analyzed in this study are included in the publication article and its additional information files. All code and data used in this study are made freely available for re-use. The bioinformatic analysis code is available as a GitHub repository (https://github.com/EngqvistLab/yeast_xylanase) and the main output files, with information on predicted CAZyme domains, are downloadable from Zenodo (https://zenodo.org/deposit/4548336; https://doi.org/10.5281/zenodo.4548336).

## Abbreviations

AX: Arabinoxylan
CAZyme: Carbohydrate active enzyme
CBM: Carbohydrate-binding module
CE: Carbohydrate esterase
CMC: Carboxymethyl cellulose
CBP: Consolidated bioprocessing
DNS: Dinitro salicylate
GalMan: Galactomannan
GH: Glycoside hydrolase
GluMan: Glucomannan
GT: Glycoside transferases
GX: Glucuronoxylan
PL: Polysaccharide lyase
poly-MeGal: Poly-methylgalacturonan
SDS-PAGE: Sodium dodecyl sulphate-polyacrylamide gel electrophoresis
