# Supplementary material for: CAZyme prediction in ascomycetous yeast genomes guides discovery of novel xylanolytic species with diverse capacities for hemicellulose hydrolysis
Source: Biotechnol Biofuels. 2021 Jul 2;14:150. doi: 10.1186/s13068-021-01995-x (PMC8254220; doi:10.1186/s13068-021-01995-x)
Supplement: Supplementary file 1 — Additional file 1: Table S1. Table S1. CAZyme families grouped by polysaccharide degradation function for heatmap generation. [file 13068_2021_1995_MOESM1_ESM.pdf]

Table S1. CAZyme families grouped by polysaccharide degradation function for heatmap generation.

Beta-glucan	Cellulose	Chitin	Lignin	Mannan	Pectin	Starch	Xylan	Xyloglucan
GH3	AA3	AA11	AA1	GH2*	PL1	GH13	GH3	GH2
GH16	AA9	GH7*	AA2	GH5	PL3	GH15	GH5	GH3
GH55	GH1	GH8	CE1	GH26	PL4	GH31	GH10	GH5
	GH3	GH16*	CE15	GH27	PL26	GH63	GH11	GH12*
	GH5	GH18*		GH76	GH28	CBM21	GH30	GH16*
	GH7	GH20		GH125	GH78		GH43	GH29
	GH8	GH46*		GH134	GH106		GH51	GH31
	GH10P*	GH75			GH139		GH62	GH35
	GH12	CE4			CE8		GH67	GH42
	GH26*	CBM18					GH115	GH45
	GH45	CBM19					CE1	GH74
	GH51*	CBM32					CE3	GH95
	GH74*	CBM50					CE4	
	CBM1						CE5	
							CE12	
							CE15	
							CE16*	
							CBM13	

AA= auxiliary activity; CE=carbohydrate esterase, CBM= cellulose binding motif; GH= glycoside hydrolase; PL= polysaccharide lyases.
*****minor specificity within family.
