# Supplementary material for: CAZyme prediction in ascomycetous yeast genomes guides discovery of novel xylanolytic species with diverse capacities for hemicellulose hydrolysis
Source: Biotechnol Biofuels. 2021 Jul 2;14:150. doi: 10.1186/s13068-021-01995-x (PMC8254220; doi:10.1186/s13068-021-01995-x)
Supplement: Supplementary file 2 — Additional file 2: Fig. S1. SDS-PAGE gel showing proteins secreted by B. mokoenaii after three days of growth in xylan containing Delft medium. [file 13068_2021_1995_MOESM2_ESM.pdf]

Additional file 2, figure S2

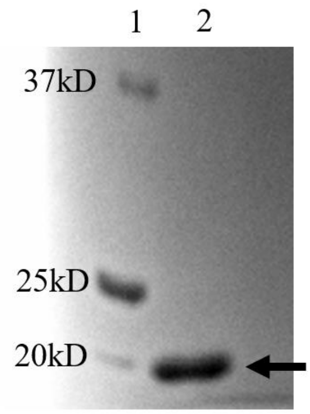

Fig. S2. SDS-PAGE gel showing proteins secreted by *B. mokoenaii* after three days of growth in xylan containing Delft medium. A GH11 xylanase at 23.19 kDa is indicated by a black arrow. Lane 1 = molecular weight ladder, lane 2 = *Blastobotrys mokoenaii* concentrated secretome.
