# Supplementary material for: CAZyme prediction in ascomycetous yeast genomes guides discovery of novel xylanolytic species with diverse capacities for hemicellulose hydrolysis
Source: Biotechnol Biofuels. 2021 Jul 2;14:150. doi: 10.1186/s13068-021-01995-x (PMC8254220; doi:10.1186/s13068-021-01995-x)
Supplement: Supplementary file 3 — Additional file 3: Fig. S2. Phylogenetic analysis of GH11 and GH10 xylanases. [file 13068_2021_1995_MOESM3_ESM.pdf]

**Additional file 3. Fig. S3.**

**a**

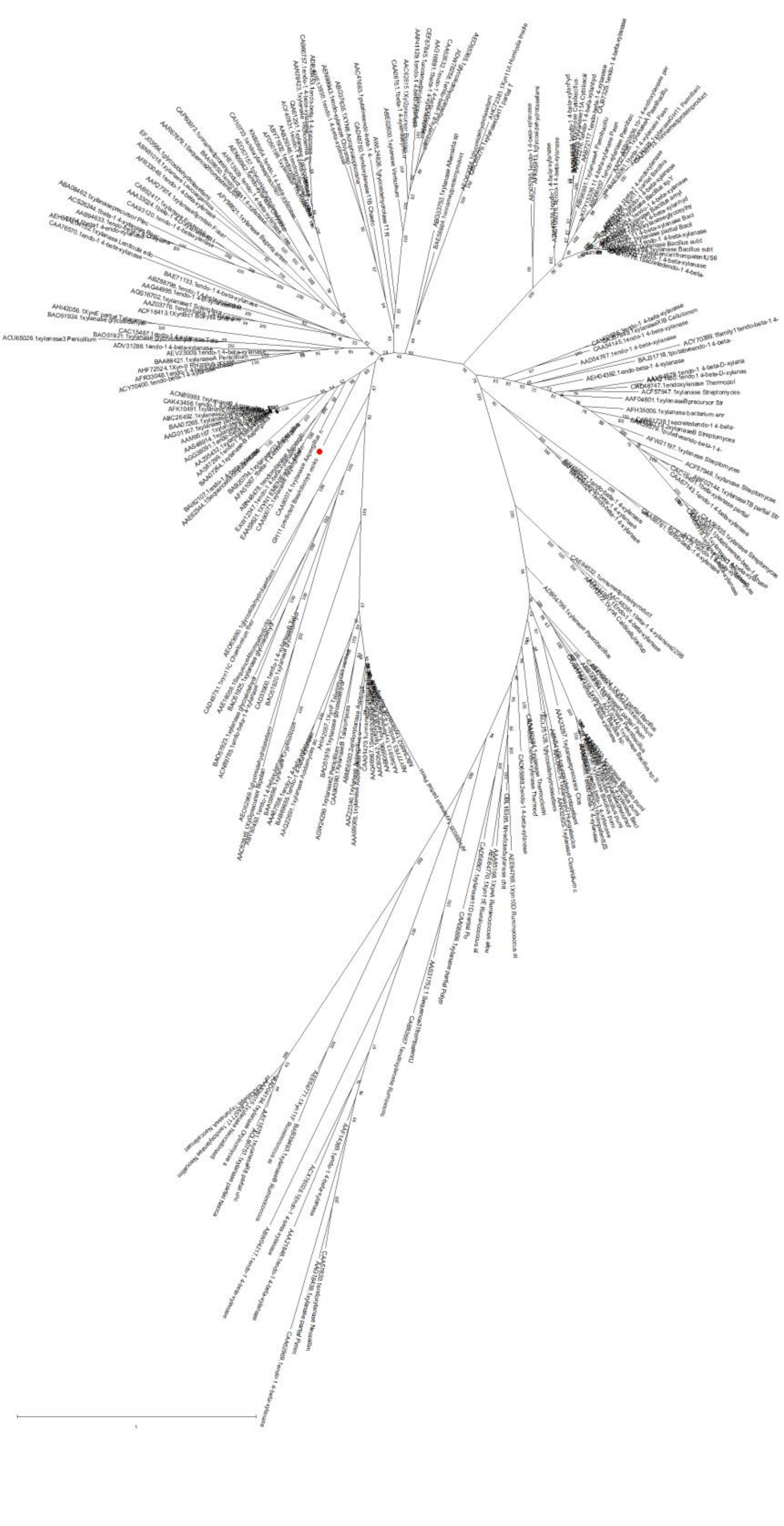

**b**

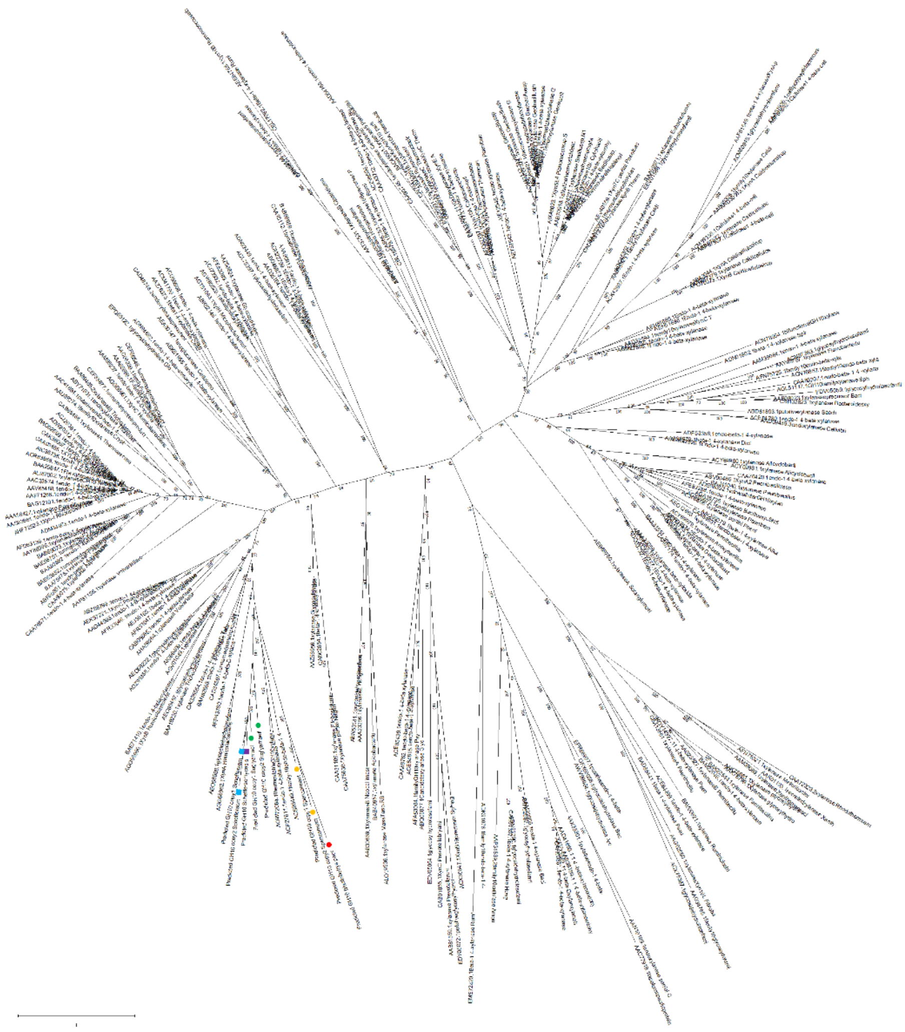

**Fig. S3.** Phylogenetic analysis of GH11 and GH10 xylanases. **a** Phylogenetic placement of the GH11 xylanase from *B. mokoenaii* (Red cirlce) and **b** Phylogenetic placement of the GH10 xylanases from *Sp. europaea* (yellow circles), *Su. lignohabitans* (green circles), *B. peoriensis* (red circle), *Sc. lignosu*s (blue squares) and *Sc. stipitis* (purple square). The molecular phylogenetic analysis was performed using full protein sequences from 259 GH10 and 208 GH11 characterized enzymes using Newick tree model from MUSCLE alignment with 1000 boot strap replicates. The numbers at each branch indicate bootstrap values and tree topology confidence. Trees are drawn to scale with branch lengths measured in numbers of substitutions per site. Scale bars represents 1.0 substitutions per nucleotide position.
